# Antenna Systems and Electronic Warfare Applications. Edited by Richard A. Poisel, Artech House, 2012; 1036 pages. Price: £129.00, ISBN 978-1-60807-484-6

**DOI:** 10.3390/s130101158

**Published:** 2013-01-17

**Authors:** Shu-Kun Lin

**Affiliations:** MDPI AG, Kandererstrasse 25, CH-4057 Basel, Switzerland; E-Mail: lin@mdpi.com

The following paragraphs are reproduced from the website of the publisher [1].

This comprehensive book serves as a one-stop resource for practical EW antenna system know-how. Supported with over 700 illustrations and nearly 1,700 equations, this authoritative reference offers you detailed explanations of all the important foundations and aspects of this technology. Moreover, you get an in-depth treatment of a wide range of antenna system applications.

The book presents the key characteristics of each type of antenna, including dipoles, monopoles, loops, arrays, horns, and patches. This authoritative volume enables you to analyze and design broadband communication and radar EW antennas, interface antennas to receivers and power amplifiers with maximum efficiency, use multicouplers to connect multiple receivers to a single antenna, and apply the correct kind of antenna to EW problems.

**Table of Contents****Preface****Chapter 1. Introduction to electronic warfare antenna systems****Chapter 2. Principles of electromagnetic radiation****Chapter 3. Fundamental antenna properties****Chapter 4. Transmission lines****Chapter 5. Dipole antennas****Chapter 6. Monopole antennas****Chapter 7. Loop antennas****Chapter 8. Traveling wave antennas****Chapter 9. Antenna arrays****Chapter 10. EW applications of antenna arrays****Chapter 11. Yagi-Uda antennas****Chapter 12. Frequency independent EW antennas****Chapter 13. Aperture antennas****Chapter 14. Electrically small EW antennas****Chapter 15. Patch antennas****Chapter 16. EW applications of patch antennas****Chapter 17. Adaptive EW antenna arrays****Chapter 18. Fractal antennas****Chapter 19. Genetically designed EW antennas****Chapter 20. Antenna matching****Chapter 21. Multicouplers, combiners, and diplexers****Chapter 22. Radomes****Appendix A. RF amplifiers****Appendix B. RF switches****Appendix C. The method of moments****Appendix D. Properties of dielectric materials.****List of Acronyms****About the Author****Index**
